# IDEPI: Rapid Prediction of HIV-1 Antibody Epitopes and Other Phenotypic Features from Sequence Data Using a Flexible Machine Learning Platform

**DOI:** 10.1371/journal.pcbi.1003842

**Published:** 2014-09-25

**Authors:** N. Lance Hepler, Konrad Scheffler, Steven Weaver, Ben Murrell, Douglas D. Richman, Dennis R. Burton, Pascal Poignard, Davey M. Smith, Sergei L. Kosakovsky Pond

**Affiliations:** 1 Interdisciplinary Bioinformatics and Systems Biology Program, University of California San Diego, La Jolla, California, United States of America; 2 Department of Medicine, University of California San Diego, La Jolla, California, United States of America; 3 Department of Pathology, University of California San Diego, La Jolla, California, United States of America; 4 San Diego Veterans Affairs Healthcare System, San Diego, California, United States of America; 5 The Scripps Research Institute, La Jolla, California, United States of America; 6 Ragon Institute of MGH, MIT, and Harvard, Boston, Massachusetts, United States of America; Carnegie Mellon University, United States of America

## Abstract

Since its identification in 1983, HIV-1 has been the focus of a research effort unprecedented in scope and difficulty, whose ultimate goals — a cure and a vaccine – remain elusive. One of the fundamental challenges in accomplishing these goals is the tremendous genetic variability of the virus, with some genes differing at as many as 40% of nucleotide positions among circulating strains. Because of this, the genetic bases of many viral phenotypes, most notably the susceptibility to neutralization by a particular antibody, are difficult to identify computationally. Drawing upon open-source general-purpose machine learning algorithms and libraries, we have developed a software package IDEPI (IDentify EPItopes) for learning genotype-to-phenotype predictive models from sequences with known phenotypes. IDEPI can apply learned models to classify sequences of unknown phenotypes, and also identify specific sequence features which contribute to a particular phenotype. We demonstrate that IDEPI achieves performance similar to or better than that of previously published approaches on four well-studied problems: finding the epitopes of broadly neutralizing antibodies (bNab), determining coreceptor tropism of the virus, identifying compartment-specific genetic signatures of the virus, and deducing drug-resistance associated mutations. The cross-platform Python source code (released under the GPL 3.0 license), documentation, issue tracking, and a pre-configured virtual machine for IDEPI can be found at https://github.com/veg/idepi.

This is a *PLOS Computational Biology* Software Article

## Introduction

The challenge of predicting a viral phenotype from sequence data has many motivating examples in HIV-1 research. In this work we restrict our attention to predicting binary phenotypes, e.g. resistant *vs* susceptible, although IDEPI can be extended to predict continuous phenotypes as well. Perhaps the most established application is that of determining whether or not the viral population in a particular host harbors drug resistance associated mutations (DRAMs) [1]. Algorithms for inferring this from viral genotype alone (e.g. [2]) are well established and used both in research [3] and in clinical practice [4]. These algorithms have been developed based on large training sets using phenotypic assays, for example those measuring half maximal inhibitory concentration (IC_50_) of an antiretroviral drug (ARV) [5] to label sequences resistant or susceptible. For many ARVs, the genetic basis of resistance is simple and consists of specific point mutations [1]. This makes it possible to distinguish resistant viruses from their susceptible counterparts by the presence or absence of a specific residue or a set of residues, leading to reliable prediction [6], [7]. For other ARVs, including some protease, integrase, nucleoside reverse transcriptase inhibitors, and co-receptor antagonists, the resistance phenotype is determined by the interaction of many sites [8]–[12], or the protein tertiary structure [13], [14], prompting ongoing methodological development (e.g. [15]–[17]).

Another popular prediction problem is that of determining which of the two cellular co-receptors needed for HIV-1 fusion with (and infection of) the target cell can be used by a particular viral strain. The ability of a virus to bind CCR5 (R5-tropic), CXCR4 (X4-tropic), or either (dual-tropic) determines the efficiency with which it can infect different types of target cells [18], predicts whether or not certain ARVs will be effective [19], and impacts the course of disease progression [20]. The primary determinant of co-receptor usage is thought to be the third variable loop (V3) of the envelope glycoprotein (*env*) [21], which spans approximately 35 amino-acid residues. Specialized assays can be used to determine the tropism of a virus with a particular *env* protein [22], providing both the training sets and the gold standard against which computational prediction methods can be compared [23], [24]. Starting with the work by Fouchier and colleagues in 1992 [25], which used the computed total charge of V3 to derive and experimentally validate the simple 11/25 rule (if residues at sites 11 and 25 are positively charged, then the virus is classified as X4 tropic), numerous authors have applied decision trees [26], random forests [27], position-specific scoring matrices [28], support vector machines (SVM) [26], neural networks [29], Bayesian networks [30], and hybrid models [31] to the problem. Various feature engineering approaches including using structural information [32], electrostatic hulls [27], sequence motifs [28], and positional and segment residue frequencies [31] have also been attempted. At present the best methods achieve accuracy on the order of 85% on comprehensive training datasets, thereby justifying ongoing research to improve this value [33].

A different class of prediction problems arises naturally when researchers seek to infer genetic "signatures" of HIV-1 isolates from different anatomical compartments (e.g. blood vs cerebro-spinal fluid [34]), individuals with different clinical attributes (e.g. those with and without neurocognitive impairment [35]), and different disease stages (e.g. acute vs chronic infection [36]). Once again, the interest is both in prediction for unlabeled sequences, for example to modify treatment before impairment occurs [35], and in finding predictive features, for instance to target vaccine research towards HIV-1 strains that are more likely to establish new infections [36].

One of the most promising avenues of HIV-1 vaccine research provides our final example of genotype to phenotype association problems, and the one that IDEPI was specifically developed to address. Rational HIV-1 vaccine design has been greatly advanced by the isolation and identification of broadly neutralizing antibodies (bNab), typically from chronically infected individuals [37]. By definition, a bNab is able to neutralize (in experimental assays) a large proportion of reference viruses (e.g. [38]–[40]). Understanding which epitopes are being targeted can reveal "conserved" elements shared by many circulating viruses, and help design a vaccine which elicits responses to the same epitopes [41]. While powerful and illuminating, current biochemical and structural techniques for mapping bNab epitopes (e.g. [39], [40], [42]), are expensive, time consuming, and do not necessarily lead to good predictive models (e.g. [43]). The appeal of computational epitope prediction lies in generating hypotheses for experimental validation and in high-throughput screening of sequences with unknown resistance phenotypes. As a byproduct of bNab characterization, large panels of phenotypic (IC_50_) and matched envelope sequences have been generated, and several recent efforts [44]–[48] have been directed at applying machine learning techniques to these data in order to predict the resistance phenotypes of HIV-1 strains and to infer antibody epitopes.

To provide a unified solution for these and similar problems, we designed IDEPI – a domain-specific and extensible software library for supervised learning of models that relate genotype to phenotype for HIV-1 and other organisms. IDEPI makes use of open source libraries for machine learning (scikit-learn, scikit-learn.org/), sequence alignment (HMMER, hmmer.janelia.org/), sequence manipulation (BioPython, biopython.org), and parallelization (joblib, pythonhosted.org/joblib), and provides a programming interface which allows users to engineer sequence features and select machine learning algorithms appropriate for their application.

IDEPI is *powerful and accurate*: when we compare its performance with that of specialized tools on the four classes of problems outlined above, we find that even without feature and machine learning method tuning, IDEPI closely hews to or even outperforms existing methods on the same data. IDEPI infers *biologically meaningful features*: for each studied problem IDEPI identified many or most of the genetic sequence features that have been previously shown to affect phenotype. IDEPI is *convenient*: by standardizing data manipulation, e.g. aligning sequences to standard reference coordinates, extracting features to be modeled, reading and handling phenotype annotation, and providing means to save learned models and easily reuse them later, IDEPI can empower researchers interested in tackling new problems to focus on innovation, instead of rote utility software development; IDEPI makes tasks like retraining a classifier on different data sets trivial – something that is difficult to impossible to do with many published algorithms. IDEPI is *fast*: automatic parallelization of independent tasks (e.g. cross-validation) on multi-core architectures greatly accelerates model learning and performance evaluation; for the default linear support vector machine (LSVM) classifier, classification of new sequences given a model can be done at a rate of 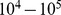 sequences per minute, making the program suitable for the analysis of next generation sequencing data. IDEPI is *customizable*: different machine learning algorithms implemented in scikit-learn can be used; new sequence features can be defined using a well-specified application programming interface (API); various feature selection approaches (e.g. forward or backward selection) can be used; performance can be optimized with respect to many metrics (e.g. sensitivity).

## Design and Implementation

### IDEPI architecture and dependencies

IDEPI is implemented in the Python 3 programming language and leverages open-source and community-developed libraries to implement reusable functionality: BioPython for biological sequence data structures and for parsers of FASTA- and Stockholm-format files; NumPy (numpy.org) and SciPy (scipy.org) for vector, matrix, and other common numerical recipes; and scikit-learn (scikit-learn.org) for various machine-learning algorithms. When extending the facilities provided by these libraries, IDEPI provides compatible application programming interfaces so that its components are reusable and similarly extensible.

**Figure 1:**
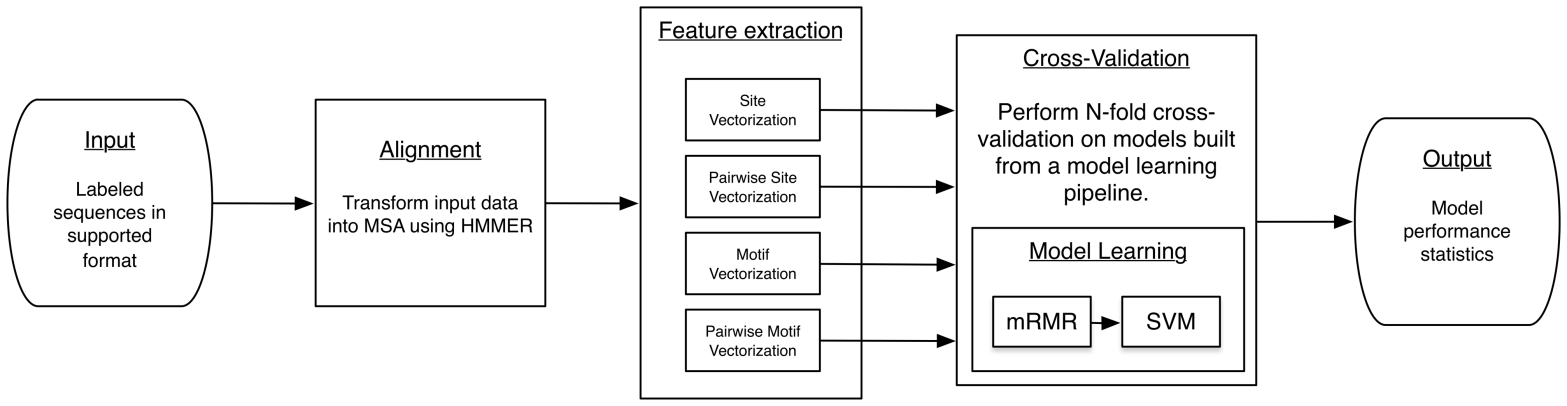
IDEPI workflow. Abbreviations: MSA - multiple sequence analysis; mRMR - minimum redundancy maximum relevance; SVM - support vector machine.

IDEPI accepts two forms of input data – a specially-crafted SQLite database (sqlite.org) or a combination of FASTA-formatted sequences with supplemental phenotypic data in comma-separated value (CSV) format (see Figure 1). These input data are transformed by IDEPI into a multiple sequence alignment (MSA) using HMMER (version 3.1b1). Because the authors of HMMER recommend providing amino-acid sequences to the program, IDEPI will by default translate the input sequences if they are determined to have a DNA alphabet. A user-provided reference multiple sequence alignment (MSA) is modeled by HMMER to guide an iterative construction of an MSA from the input data. IDEPI can also be instructed to treat the input MSA as fixed if automated alignment is not desired (e.g. for difficult to align sequence regions). Additionally, IDEPI includes a user-provided reference sequence in the alignment to label the columns of the MSA in a conventional manner (e.g. N332 for an asparagine at site 332). IDEPI distribution includes the standard HXB2 (genbank accession number K03455) reference sequence for assigning HIV-1 coordinates.

### Feature extraction techniques included with IDEPI

For feature extraction, IDEPI provides four classes (all scikit-learn compatible) for the vectorization of labeled MSAs.

1. Presence of a particular residue at a given site (e.g. N301N); optionally a match is returned if the residue belongs to a predefined class of biochemically similar residues, e.g. using Stanfel encoding, N301[DENQ] [49]. IDEPI can generate either amino-acid or nucleotide sequence features, with the preference specified as a command line argument (encoding).
2. Presence of a pair of specific residues at two sites, e.g. N301N+S334S, also optionally supporting class membership. To limit the number of all pairwise combinations IDEPI only considers pairs of sites that are no more than 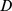 (a user-tunable parameter) positions apart in the linear sequence.
3. Presence of a sequence motif defined by a regular expression, e.g. potential N-linked glycosylation sites (PNGS), using the regular expression "N[∧P][TS][∧P]".
4. Presence of a sequence motifs defined by the same regular expression at two sites at once, e.g. PNGS (N234+N276).

For label extraction, IDEPI provides a class which converts phenotype data to a form usable by scikit-learn.

### Feature selection and learning algorithms used by IDEPI

To enable rapid learning and prevent overfitting, IDEPI performs feature selection using the minimum redundancy maximum relevance (mRMR) algorithm [50]. Briefly, the algorithm chooses features sequentially (the greedy approach), in way to maximize the mutual information with the label and minimize mutual information with already-chosen features. Sets of strongly correlated features will be typically represented by single member in the model selection process. IDEPI provides a mechanism to report all "similar" features, so that possible biological features are not masked by accidental correlates. mRMR is implemented in the separate sklmrmr package, also scikit-learn compatible, and uses Cython (cython.org) for high performance.

Default model learning is implemented using a soft-margin, linear support vector machine. The soft-margin parameter, 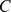, is chosen by (inner) grid search to maximize a performance metric chosen by the user (Matthews Correlation Coefficient is the default). Both of these functions are implemented within scikit-learn, and parallelized when possible.

### Tools included with IDEPI

IDEPI provides three scripts for end users not wishing to directly program their own pipelines.

1. “idepi discrete” accepts labeled sequence data and will: generate an MSA from these data, extract features and labels, perform N-fold cross-validation on models built from a pipeline of mRMR and soft-margin linear SVMs, and finally report the models' performance along with the labels of the most frequently selected features and their relationship to the models (e.g. is the presence or absence of the feature indicative of an outcome).
2. “idepi learn” will similarly accept labeled sequence data, learn a model, and save it to disk for later use.
3. “idepi predict” accepts a saved model and some unlabeled sequences (homologous to the model) and will predict their labels.

All the results presented in the manuscript have been generated using these three scripts, and detailed tutorials are available at http://github.com/veg/idepi.

### Extensible API for feature engineering

IDEPI defines a “LabeledMSA” class as a wrapper around BioPython's “MultipleSeqAlignment” for the column-wise labeling of an MSA. Together with classes facilitating alphabet encoding, IDEPI provides simple facilities enabling rapid feature engineering for biological sequence data. Examples of how these facilities can be used can be found within IDEPI 's source code – the “SiteVectorizer” and “MotifVectorizer” classes for feature extraction. Additionally, motif features are trivially supported by the “MotifVectorizer” class, which accepts a regular expression argument describing the motif. IDEPI uses this functionality to extract features for potential N-linked glycosylation sites (PNGS), using the regular expression described above.

## Results

We first tested IDEPI on simulated data and on well-studied problems of drug-resistance and tropism prediction and detection of tropism-associated genetic features. The large number of published methods make a comprehensive comparison infeasible, hence we selected methods based on their popularity, recency, performance, and the availability of training data. IDEPI was evaluated for (i) its performance in phenotype prediction using standard cross-validation metrics and on previously published independent datasets; and (ii) the veracity of the genetic features inferred to be informative of a particular phenotype. All the datasets and instructions needed to run them with IDEPI are provided with the package distribution.

### Simulated data

In order to establish baseline performance of IDEPI where the true "phenotype" is known, we simulated the evolution of 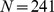 HIV-1 protein envelope sequences subject to directional selective pressure applied to sites in an epitope along a subset of terminal tree branches selected at random. For this moderate size data set (chosen to represent a typical bNab training set), IDEPI performs very well overall (Table 1), both in terms of classification performance and in recovering the locations/residue identity of epitopes. In the simplest case, when any mutation in a 5-site epitope confers resistance, IDEPI delivers a Matthews Correlation Coefficient (MCC) of 0.98 (MCC of 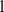 indicates a perfect classifier, and MCC of 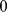 corresponds to "no-better than random prediction" performance), and recovers 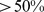 of sites within epitopes if they are sufficiently variable. Because positions in epitopes are likely quite correlated, mRMR redundant feature selection captures essentially all of the signal with a median of 2 features per replicate. For a fixed training data set size, with the increased epitope length and complexity, the performance degrades predictably, but MCC remains excellent for intermediate (8 sites and 2 or more mutations needed for escape) epitope complexity (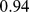) and good (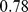) for high (10 sites and 3 or more mutations needed for escape) epitope complexity. Encouragingly there seems no false association signal due to the phylogenetic relatedness of the samples: IDEPI yields a median MCC of 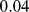 for randomly assigned phenotypes, which is essentially the same as a random prediction (also see discussion of the 2F5 bNab below).

**Table 1:** IDEPI performance in predicting phenotype and recovering features from simulated data.

Simulation	L	M	Median performance metrics, phenotype				Mean epitope recovery, by class			
			Sensitivity	Specificity	MCC	Features	Slow, %	Intermediate, %	Fast, %	FP
Simple	5	≥1	0.98	1.0	0.98	2	11.1	56.6	80.0	0.09
Intermediate	8	≥2	0.95	1.0	0.94	3	10.4	42.6	71.6	0.16
Complex	10	≥3	0.85	0.98	0.78	3	6.0	39.4	58.3	0.16
Random	N/A	N/A	0.57	0.47	0.04	1	N/A	N/A	N/A	1

Forward feature selection (to optimize MCC), and 10-fold nested cross-validation were used to learn the models. **L**: the number of sites in an epitope; **M**: how many escape mutations are needed to confer resistance; epitope recover classes are based on simulated evolutionary rates; **FP**: mean (per replicate) number of selected features not in a simulated epitope; a feature was counted as recovered if it were selected in 50% or more of cross-validation replicates.

### Drug resistance

We used a large publicly available data set of viral sequences (reverse transcriptase) and matched IC_50_ values for the PhenoSense assay (available from the Stanford HIV Drug Resistance Database, hivdb.stanford.edu) to train an IDEPI classifier for resistance to a non-nucleoside reverse transcriptase inhibitor nevirapine (NVP). We chose this drug as a test case because (i) the basis for its resistance is well understood, making the assessment of IDEPI predictions easy; (ii) testing for NVP resistance is biomedically relevant, for example in the context of preventing mother to child HIV-1 transmission; (iii) a recent study [51] used resistance data from the Stanford database to train specialized classifiers for NVP resistance, providing a basis for comparison.

**Figure 2:**
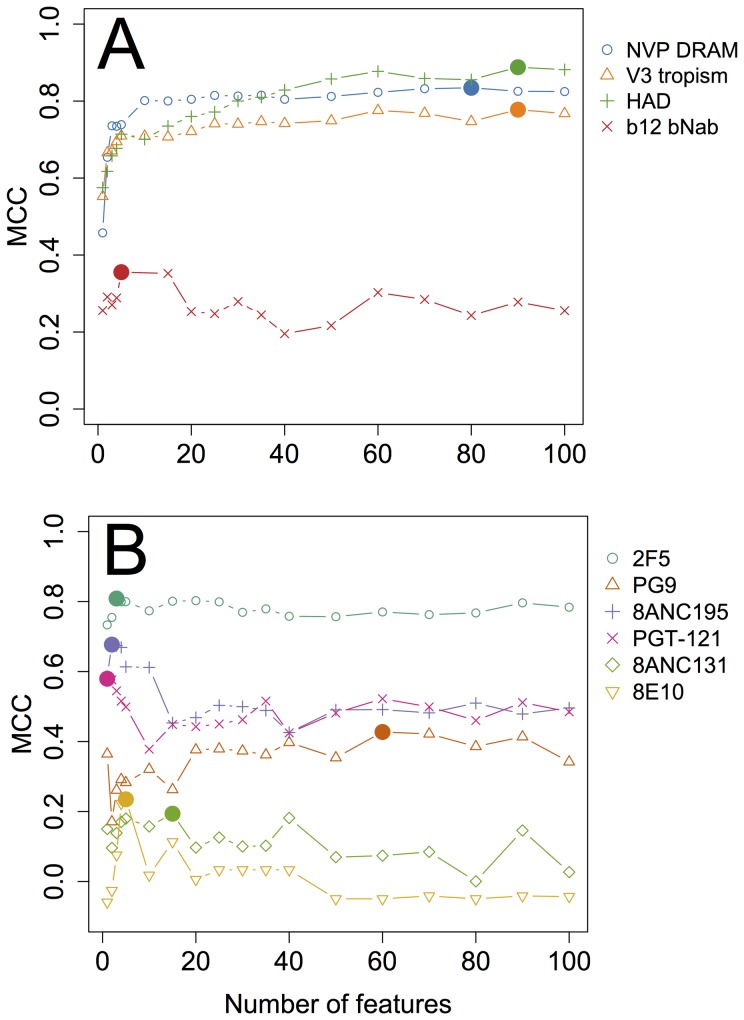
IDEPI performance, measured by MCC, as a function of the number of model features. (A): on a representative of each of the four classification problems, (B): on predicting resistance to a particular broadly neutralizing monoclonal antibody. Abbreviations: NVP - Nevirapine; DRAM - drug resistance associated mutations; HAD - HIV associated dementia; bNab - broadly neutralizing antibody. The optimal number of features is highlighted with a filled circle for each line plot.

With 80 features (the number selected by a grid search, see Figure 2.A), IDEPI achieves the same accuracy (0.92, Table 2) as a state-of-the-art custom-built prediction tool using structural information [51]. The first three selected features (K103K, Y181Y, G190G, see Table 3), correspond to three canonical sites of strong phenotypic resistance, and the maintenance of the wildtype residue at each of the positions is strongly predictive of susceptibility – a classifier built on just these three features achieves an MCC of 0.74, compared to that of 0.83 for the 80-feature model. Other genetic features implicated in the development of NVP resistance recovered by the IDEPI model include major resistance mutations K101P, K103N, V106A, Y181C, Y188L, G190A, and accessory/weak resistance mutations L100I, E138Q, H221Y, and V108V, P236P (the latter two associated with susceptibly) [1]. Note that the same site can appear in multiple features (e.g. Y181Y as a feature of susceptibility and Y181C – as a feature of resistance), hence an 80-feature model does not span 80 different sites of HIV-1 reverse transcriptase.

**Table 2:** IDEPI performance in predicting phenotypes from genotypes based on training data analyzed previously.

Problem	N	B	F	IDEPI performance				
				5-fold cross-validation metrics				Benchmark (IDEPI: ref)
				Sens.	Spec.	Accu.	MCC	
NVP resistance	1461	62.3%	80	0.88	0.97	0.92	0.83	CV Accu. 0.92: 0.92^1^
V3 tropism	1356	15.1%	90	0.89	0.94	0.94	0.78	Training Accu. 0.95:0.96^2^
Dementia	861	70.3%	90	0.96	0.93	0.95	0.89	CV Accu. 0.95:0.75^3^
2F5 bNab	465	48.6%	3	0.93	0.88	0.90	0.81	Training Accu. 0.90 vs proportion of residuals explained 0.49^4^
b12 bNab	247	64.4%	5	0.74	0.62	0.70	0.36	Training Accu. 0.75:0.86^5^
10E8 bNab	178	4.0%	5	0.30	0.96	0.93	0.23	Training Accu. 0.96 vs proportion of residuals explained 0.21^5^
PG9 bNab	301	26.2%	60	0.56	0.86	0.78	0.43	Training Accu. 0.96 vs proportion of residuals explained 0.31^5^
PGT-121 bNab	118	37.2%	1	0.80	0.79	0.80	0.58	Training Accu. 0.80 vs proportion of residuals explained 0.52^5^
8ANC131 bNab	178	30.9%	15	0.51	0.69	0.63	0.19	
8ANC195 bNab	178	42.7%	2	0.94	0.75	0.83	0.67	Training Accu. 0.83 vs proportion of residuals explained 0.58^5^

IDEPI metrics were obtained using 5-fold cross-validation. B (balance) is defined as the proportion of "positive" training samples. The number of features (F) was chosen by selecting a value from a pre-defined grid to maximize cross-validation MCC.

1 random forests trained on combined sequence and structural features using resistance classifications from the Stanford Drug Resistance Database [51];

2 a two-level classifier combining random forest predictions based on an electrostatic hull and hydrophobicity features of the V3 loop (680 features) trained on the same data [27];

3 a hierarchical decision tree classifier using composite amino-acid features trained on the same data [35].

4 a rule based additive regression model trained to minimize IC_50_ residuals [45].

5 an ensemble classifier using signature rules and logistic regression trained on the same data [44].

**Table 3:** Key features selected by IDEPI for each of the example problems.

Problem	Features selected by IDEPI				
	Rank	Identity	Direction	MCC	Remarks
NVP resistance	1	K103K	Susceptible	0.46	Canonical NNRTI resistance site [1]
	2	Y181Y	Susceptible	0.65	Canonical NNRTI resistance site
	3	G190G	Susceptible	0.74	Canonical NNRTI resistance site
V3 tropism	1	PNGS(N301)	CCR5	0.55	Essential for CCR5 binding [55] and
	2	R306R	CCR5	0.67	dual-tropic viruses [67] Part of the 11/25 rule [25]
Dementia	1	T297K	Non-HAD	0.57	
	2	PNGS (N488)	HAD		
	3	R298D	Non-HAD		
	4	I320[]	non-HAD		
	5	PNGS(T188)	HAD	0.71	
2F5 bNab	1	K665K	Susceptible	0.73	Parts of the canonical
	2	A667A	Susceptible	0.75	linear epitope (662–667) [59]
b12 bNab	1	D185D	Susceptible	0.26	The strongest association found in
					[44]
10E8 bNab	3–4	T676T	Susceptible	N/A	A part of the structural epitope [64]
PG9 bNab	1	PNGS (N160)	Susceptible	0.36	Key residue for binding based on
	8	V169E	Resistant		structure [62] Forms a hydrogen bond with PG9
					[62]
PGT-121 bNab	1	PNGS(N301+N332)	Susceptible	0.58	tralization [63]
8ANC195 bNab	1	PNGS (N234+N276)	Susceptible	0.59	Encompasses the three mutants (sites 234, 236, and 276) any of which confers resistance [45], [46] PNGS at site 230 confers weak resis-tance [45]
	2	PNGS(N160+N230)	Resistant	0.67	
8ANC131 bNab	3.75	PNGS(N339+Q442)	Resistant		
	5	K151G	Susceptible		

Notation: T297K means that K is found in position 297 (HBX2 coordinates, T is the residue found in HXB2); PNGS (T188) – a potential N-linked glycosylation site with N at HXB2 coordinate 188; PNGS (N234+N276) – a pair of potential N-linked glycosylation site with N at HXB2 coordinates N234 and N276; [] – a deletion relative to HXB2. The ranking of the features is based on what order they were added to the model, and averaged over cross-validation replicates. For datasets with little signal (e.g. 10E8 bNab, 8ANC131 bNab), there was considerable variation in feature ranks among CV replicates, hence the best ranking feature has a median rank worse than 1. The values in the MCC column are for the models with the corresponding number of features (e.g. the MCC of a 2-feature model for V3 tropism in 0.67).

We compared the SVM model learned by IDEPI against perhaps the most commonly used drug resistance prediction algorithm – the Stanford HIVdb (expert curated, and evidence based) [2], using a large dataset collected in Mexico for the purposes of drug resistance surveillance [52]. Because no phenotype measurements were available for these sequences (as is common in practice), we computed the degree of concordance between IDEPI and HIVdb using Cohen's 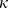 [53]. Since HIV-1 pol sequences obtained during routine surveillance are amplified from mixed viral populations and often contain ambiguous bases, not directly handled by default IDEPI feature sets, we considered all possible amino-acid resolutions of nucleotide level ambiguities at the positions involved in the model, and called a sequence resistant if any of the resolutions were predicted as resistant. The two methods of resistance prediction were in excellent agreement overall (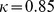), including all cases of "highly-resistant" sequences. This is on par with the numbers reported in a recent comparison of several rule-based resistance prediction algorithms [54].

### Co-receptor usage/tropism

In 2010, Dybowski et al [27] presented a sophisticated multi-level classifier including structural and biochemical properties of the V3 loop, performed extensive training and validation of their approach, and compared it to previous work. Because a large training data set of V3 amino-acid sequences and associated phenotypic measurements was provided as a part of the publication, we were able to train an IDEPI classifier on the same data to enable a direct comparison.

As has been documented before (e.g. [27]), most of the predictive power of V3 sequences is captured by only a few features – in the case of IDEPI, a model using only two features already achieves an MCC of 0.67, while the full model with 90 features improves it to 0.78. The first selected feature is a potential N-linked glycosylation site (PNGS) at position 301; several sites in this 4-residue motif have been implicated as critical to CCR5 receptor binding [55], hence a single composite feature is able to encapsulate the discriminating positions for many sequences. The second feature is one of the two residues in the 11/25 rule [25]; interestingly, the two positions are sufficiently correlated in the training sample that mRMR feature selection eliminates position 25 once 11 has been included. IDEPI appears to be surprisingly well suited to the problem of tropism prediction, and delivers nearly the same accuracy (0.94 vs 0.96, the latter number obtained in the original publication by tuning algorithmic cutoffs to maximize accuracy on the training data) as the much more complex feature engineering approach undertaken by Dybowski and colleagues. Furthermore, on an independent dataset, IDEPI attains accuracy of 0.905, whereas the best of the 5 methods compared previously [27] attained accuracy of 0.86.

### HIV-1 associated dementia

A recent comprehensive study by Holman and Gabuzda [35] applied a machine learning pipeline (based on decision trees) to partial envelope sequences to identify signatures (defined as collections of residues or biochemical properties at specific genomic positions) of sequences isolated from brain tissue of subjects who developed HIV-1 associated dementia (HAD). Since the training set of sequences and corresponding diagnoses has been kindly made available by the authors through the HIV Brain Sequence Database [56], it was straightforward to apply IDEPI to the same data to learn a classifier. The Holman and Gabuzda study also included an independent validation data set of 10 individuals diagnosed with HAD, and we used it here to test the learned model.

IDEPI excels at this classification problem, with both specificity and sensitivity exceeding 0.9, and achieving an accuracy of 0.95. The original authors reported an accuracy of 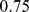, but their model was restricted to a subset of the available sequence length, HXB2 *env* amino-acid coordinates 265–369. When restricted to the same subset of residues, IDEPI achieves an accuracy of 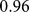 with 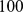 features (detailed results not shown), suggesting that many of the predictive features are correlated (and mRMR selects only one), because the performance does not degrade when only partial sequences are considered. As with previous two applications, a single prominent feature (T297K) attains an MCC of 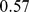; unlike the other problems, the next four features appear to be of about the same informative content (based on the order in which they are selected in cross-validation folds), and MCC performance increases gradually as the features are added (Figure 2). Interestingly, features previously reported as associated with HAD (see [35] for a summary), are not added to the model until later: for example site 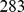 is the 8th ranked feature, site 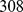 is the 38th, and site 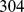 is the 65th. Furthermore, the 90-feature IDEPI correctly classifies all 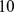 individuals in the validation data set, whereas the original method correctly classified 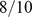 cases.

### Broadly neutralizing antibodies

Because IDEPI was designed for the specific problem of finding bNab epitopes and predicting the resistance phenotype from sequence data, we compared its performance against three recently published machine learning approaches to solving same problem.

1. Gnanakaran et al [44] proposed and tested an ensemble framework combining pattern analysis and logistic regression to predict the neutralization phenotype and map the epitopes of the b12 bNab [57], which targets the CD4 receptor binding site [58]. We used the genotypic and associated phenotypic data from this study to train and test an IDEPI classifier for the b12 bNab.
2. West et al [45] applied a direct optimization (implemented in the Antibody Database program [ADP]) to predict the continuous IC_50_ value using sequence based features and applied it to data from 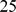 antibodies. We compared the predictions derived by IDEPI models for some of the same antibodies (chosen to represent one of the remaining three types of bNab classified by their targets [58]), using either publicly available neutralization assay data, distributed with IDEPI, or the training data set from [46].
3. Chuang et al [46] developed an epitope feature selection which evaluates various measures based on mutual information between sequence sites and IC_50_ values – an idea shared and extended by the mRMR approach. We used the genotype and phenotype data for two of the antibodies (8ANC131 and 8ANC195, the latter also studied by West et al) whose epitopes were mapped and experimentally confirmed by Chuang et al.

#### 2F5 bNab prediction

2F5 is the first characterized bNab which targets the linear Membrane-Proximal External Region (MPER) region of HIV-1 viral envelope [59]. 2F5 provides a natural baseline test case for IDEPI. On the one hand, any epitope prediction approach worth its salt must perform well on this test case: the training dataset is one of the largest available, the epitope is very well characterized [59], and the eptiope is short and linear (662–667 in the HXB2 coordinates). On the other hand, 2F5 is an excellent example of a strong "clade effect", for example it neutralizes viruses of subtype B very well, but has essentially no potency against subtype C viruses [60]. Thus, a machine learning approach could potentially learn a classification model by simply finding genetic signatures that discriminate genetically divergent HIV-1 subtypes and have little to do with antibody specificity; such behavior is clearly undesirable if one seeks to find genetic determinants of resistance. On 2F5 data which we downloaded from the LANL HIV CATNAP database (hiv.lanl.gov), IDEPI achieves the best MCC performance for all bNab examined (0.81, Table 2) with a 3-feature model, demonstrating that it can learn "easy" cases well. Not unexpectedly, the first feature selected by IDEPI is a K665K (Table 3), which is in the structurally characterized 2F5 epitope, and which alone yields the MCC of 0.73. The second chosen feature (A667A) is also in the known epitope, but it improves cross-validation MCC only to 0.75. West et al [45] identified the same two features in their model (as well as three features outside the canonical epitope). The third feature in our model is not stable i.e. it is not consistently chosen between CV replicates (e.g. T373T, K490E and E824G are chosen in some of the replicates), and does not lie in the canonical epitope. The addition of a third feature improves the sensitivity of the model (from 86.7% to 92.5%), while maintaining its specificity at 88.2%; further examination of the data indicates that the third is feature is necessary to correctly classify the small proportion of sequences with resistant phenotypes which contain the sensitive canonical epitope.

Because the current implementation IDEPI assumes that the contributions of individual features to phenotype are independent and additive, it is possible that a feature in the model is not directly involved with the phenotype but is only associated with other features that are. In this context, the *related features* report may be useful: features that are strongly associated with those already selected for the model by the mRMR algorithm are reported by IDEPI. More concretely, if feature A is predictive of phenotype, feature B is only associated with phenotype due to shared ancestry, and features A and B are themselevs strongly correlated, mRMR may choose feature B as a part of the model and eliminate A from contention, but then IDEPI would report that A is related to B. Hence, the correct interpretation of the genotypic features in the example would be "either A or B are predictive of the phenotype".

#### b12 bNab prediction

Unlike the previous three applications, b12 epitope prediction results in both a simpler model (only 5 features) and a considerably lower performance (Table 2), with an MCC of only 0.35. IDEPI achieves lower accuracy on the training data than the the ensemble method developed by Gnanakaran et al [44] (note that the original reference does not report a cross-validation value), but higher accuracy on validation data (Table 4), suggesting that the ensemble model may have been over-fitting the training data. Only a single residue (D185D, Table 3) is supported by the majority of cross-validation folds. Taken together, these results suggest that the training data set is too small (or that the IDEPI feature set is suboptimal) to reliably identify the complex structurally-defined epitope for b12. However, IDEPI outperforms a previously published method on an independent validation dataset, and its 5-feature epitope includes residue 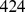 which is a part of the CD4 binding site [61] targeted by the antibody.

**Table 4:** IDEPI model performance on independent datasets and comparison with benchmark methods.

Problem	Independent dataset			
	N	Reference	Benchmark	Performance
NVP resistance	1639	[52]	Stanford HIVdb	Cohen's 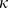 = 0.85.
V3 tropism	74	[27]	Best of 5 methods, including SVM, decision trees, and position-specific scoreing matrices [27]	Accu. IDEPI 0.91 vs 0.86
Dementia	10	[35]	Ensemble of rule learning and de-cision trees from [35]	IDEPI 10/10 vs 8/10
b12 bNab	55	[44]	Ensemble of signatures and logis-tic regression [44]	Accu. IDEPI 0.73 vs 0.61

### Other broadly neutralizing antibodies

1. **PG9** is a broadly neutralizing antibody targeting the V1/V2 loop in HIV-1 env [62], whose canonical epitope is anchored by the PNGS at position 160, which is also the single most important position in the 60-feature model fitted by IDEPI. A relatively low MCC of 0.42 is achieved, with the model showing fairly low sensitivity (0.49, Table 2). The 60 feature model has a remarkably high accuracy on the training data (0.96), but the small number of resistant sequences in it makes it difficult to generalize the features past N160 (Figure 2). A direct comparison with West et al is difficult to formulate, because the performance of ADP is measured by the proportion of IC_50_ variance explained by the model, which cannot be measured for IDEPI. IDEPI finds the three features found by ADP, but ranks them differently (more in agreement with the structural studies): G732G (resistant, ranked by ADP as having strong support, mean IDEPI feature rank 17.6/60), PNGS (N160) (susceptible, supported by structure [62], ADP: intermediate support, IDEPI: mean feature rank 7/60), and K171K (susceptible, supported by structure [62], ADP: strong support, IDEPI: no 171 feature, but a number of features in neighboring positions 170,173 and 174). Further, IDEPI places another structurally confirmed residue in the inferred epitope: V169E (resistant, mean rank 5/60), V169K (susceptible, mean rank 15/60).
2. **PGT-121** is a broadly neutralizing antibody targeting glycans in the V3 loop [40]. IDEPI infers a single feature model (Table 3), which associates the presence of a pair of PNGS (at positions 301 and 332) as strongly predictive (MCC = 0.58) of susceptibility. Interestingly, while PNGS (N332) is the key part of the canonical PGT-121 epitope, PNGS (N301) – previously thought relatively unimportant – appears to act together with N332 to effect PGT-121 binding [63]. ADP predicts the importance of PNGS (N332), but also lists four other sites whose role in antibody-virus interaction is unclear, and does not report N301 as important.
3. **10E8** is a broadly neutralizing antibody that targets the MPER region [64] and shows unusual potency versus the reference panel viruses. As a result, the training sample (Table 2) includes only 4% of resistant sequences, and this makes meaningful learning difficult, as evidenced by the low MCC of 0.23, and sensitivity of 0.30. There are no top ranked features in the model (the ranking changes significantly between cross-validations, Table 2), but one of the structurally defined epitope sites (T676T) is included among the top 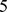, whereas ADP finds no such sites and also performs poorly. The relevance of other inferred model features associated with resistance, e.g. PNGS(T413+E824), K171E and E153Q is questionable, and larger training datasets containing more resistant samples are needed for computational prediction to improve.
4. **8ANC195** is a broadly neutralizing antibody whose epitope has not been structurally confirmed [65], but it was used as a test case for computational epitope prediction and experimental confirmation by two independent groups [45], [46]. IDEPI achieves a good MCC of 0.67 on the training data from Chuang et al, and does so with only two features in the epitope: two pairs of PNGS sites (Table 3). The top feature is that the absence of either a PNGS anchored at site 234 or a PNGS anchored at site 276 confers resistance. This single pair of PNGS subsumes three features (N234, N276, and T236) experimentally validated by previous work. This example highlights that feature engineering (pairs of PNGS) may provide a more compact description of neutralization features than either single PNGS [45], or single residues [46] can. The second feature selected by IDEPI is another pair of PNGS (N160 and N230), which is predicted to confer resistance, and does so at a weak level [45].
5. **8ANC131** is a broadly neutralizing antibody whose epitope has been structurally mapped, but not yet published [46], and the same authors performed computational prediction of epitope sites and tested them experimentally. Unlike 8ANC195, where the epitope features are clean and experimentally confirmed, computational predictions have not been found nearly as useful, with the top sites conferring only marginal resistance [46]. IDEPI finds a diffuse signal for 15 features (Figure 2, Table 2), and an MCC of 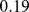. There seems to be little overlap between the features found in 3 or more cross-validation folds (susceptible: K151G, V169R, resistant: N463K, D474N, PNGS(N339+Q442), PNGS(142a+N234)), and those reported by [46] [top 10: 456,78,79,466,280,326,96,80,282,461] although many are in the same region of the three-dimensional structure.

## Availability and Future Directions

IDEPI and sklmrmr are installable via the PyPI Python package system through standard tools (easy_install/pip), and their source code is available on GitHub (github.com/veg/idepi and github.com/nlhepler/sklmrmr). A Virtual Machine for Oracle's VirtualBox has also been built to provide easy access to IDEPI for users unfamiliar with the intricacies of Python package management, and is available from the main package distribution page (http://github.com/veg/idepi/).

IDEPI will likely be extended in the future to include a larger array of built-in feature extraction mechanisms. For instance, because both amino-acid and nucleotide data can be useful for phenotype prediction (the latter could be informative about important RNA secondary structures in viruses, or transcription/translation efficiency), we will allow protein-coding sequences to be tokenized into nucleotides and amino-acids jointly. In the future, we intend to release an update that includes a feature extractor that maps sequence data to a provided structure to perform a spatial neighborhood analysis, and an adaptive discretization algorithm for continuous features (e.g. using Bayesian blocks [66]), required by mRMR. Downstream users that build novel feature extractors are recommended to submit their creations to IDEPI, via GitHub's pull request mechanism, for inclusion in a future release. Additionally, in providing APIs compatible with BioPython and scikit-learn, IDEPI will prove ever more useful as advances are made in those fast-moving software packages. Finally, we encourage those who use IDEPI and learn models using it to contribute these models by using the pull request mechanism available in GitHub. Because the models do not include original sequence data, but only HMMER models needed to make alignments, this mechanism also ensures privacy preservation of training data.

## Supporting Information

Software S1 The complete source code tree, example files, and documentation for IDEPI; for the most current version visit the package distribution page at https://github.com/veg/idepi.
(GZ) Click here for additional data file.

Text S1 Details on data simulation strategies, feature selection approaches and machine learning algorithm settings for each of the four classes of classification problems, and software library versions used for testing.
(PDF)
