# Supplementary material for: IDEPI: Rapid Prediction of HIV-1 Antibody Epitopes and Other Phenotypic Features from Sequence Data Using a Flexible Machine Learning Platform
Source: PLoS Comput Biol. 2014 Sep 25;10(9):e1003842. doi: 10.1371/journal.pcbi.1003842 (PMC4177671; doi:10.1371/journal.pcbi.1003842)
Supplement: Text S1 — Details on data simulation strategies, feature selection approaches and machine learning algorithm settings for each of the four classes of classification problems, and software library versions used for testing. (PDF) [file pcbi.1003842.s002.pdf]

# Supplementary Methods

## Simulated data

We simulated the evolution of $N = 241$ HIV-1 protein envelope sequences subject to a directional selective pressure applied to sites in an epitope using the HyPhy package [1]: the reference HXB2 sequence was evolved along a phylogenetic tree representing the diversity of circulating HIV-1 group M strains (inferred from biological isolates), subject to an HIV-1 specific substitution model [2], with site-to-site substitution rate heterogeneity modeled by a 3-bin general discrete distribution [3]. The development of resistance to a particular simulated epitope in a subset of sequences (defined as a set of positions in the genome and "escape" residue), was modeled by accelerating the rate of amino-acid substitution towards the escape residue along the terminal tree branch leading to a resistant sequence. For each replicate (100 replicates per set), an epitope of desired complexity was generated (Table 1), and each simulated sequence was assigned a phenotype as a deterministic function of its genotype. We also performed a simulation where phenotypes were assigned to sequences randomly, in order to establish the degree to which phylogenetic relatedness can drive spurious associations due to the non-independence of samples [4].

## Drug resistance

We labeled a sequence resistant to NVP if the measured fold change in $IC_{50}$ was 5 or greater. A feature was reported if it appeared in 3 or more out of 5 cross-validation replicates. We investigated the complexity of the genotypic basis of resistance by a simple grid search (the number of features was one of the following values: 1,2,3,4,5,10,15,20,25,30,35,40,50,60,70,80,90,100; see Figure 2A)

## Co-receptor usage/tropism

The number of features maximizing 5-fold cross-validation MCC was determined by a simple grid search. In addition to cross-validation performance metrics, we compared the performance of the IDEPI model to the methods considered by Dybowski et al [5] on an independent validation dataset with 74 sequences.

### Broadly neutralizing antibodies

IDEPI labeled sequences with $IC_{50}$ of $\geq 20\mu g/ml$ for a given bNab as resistant, except for the 10E8 bNab (which shows unusually low titers for the reference panel), where the threshold was lowered to $5\mu g/ml$. Because typical distributions of $IC_{50}$ values are strongly bimodal (peaks near 0 and maximum measured value of 50 $\mu$ g/ml), classification performance was not unduly sensitive to the choice of cutoff values; further, the mapping from $IC_{50}$ to phenotype labels can be specified as a run-time option, making the threshold trivially tunable. The number of features maximizing 5-fold cross-validation MCC was determined by a simple grid search (as before, the number of features was one of: 1,2,3,4,5,10,15,20,25,30,35,40,50,60,70,80,90,100).

### Computational Resources and Software Versions

All experiments were performed with IDEPI v0.17, sklmrmr v0.2.0, scikit-learn v0.14.1, scipy v0.12.0, numpy v1.7.1, BioPython v1.62, and Python v3.3.1 on a Penguin Computing Altus server (dual 8-core AMD Opteron 6128) running CentOS 6.4.

## References

1. Pond SLK, Frost SDW, Muse SV (2005) HyPhy: hypothesis testing using phylogenies. Bioinformatics 21: 676-9.

2. Nickle DC, Heath L, Jensen MA, Gilbert PB, Mullins JI, et al. (2007) HIV-specific probabilistic models of protein evolution. PLoS One 2: e503.

3. Pond SK, Muse SV (2005) Site-to-site variation of synonymous substitution rates. Mol Biol Evol 22: 2375-85.

4. Gnanakaran S, Daniels MG, Bhattacharya T, Lapedes AS, Sethi A, et al. (2010) Genetic signatures in the envelope glycoproteins of HIV-1 that associate with broadly neutralizing antibodies. PLoS Comput Biol 6: e1000955.

5. Dybowski JN, Heider D, Hoffmann D (2010) Prediction of co-receptor usage of HIV-1 from genotype. PLoS Comput Biol 6: e1000743.
